# Evidence for Early-Time Spurt-Loss Dominance in Borate-Crosslinked HPG Gel Leakoff for High-Permeability Sandstone

**DOI:** 10.3390/gels12060519

**Published:** 2026-06-10

**Authors:** Shuqian Li, Wei Liu, Beiyu Han, Jingen Deng, Liqun Li, Kaikai Xu, Liangliang Zhao

**Affiliations:** 1 Hainan Institute, China University of Petroleum (Beijing), Sanya 572000, China; lishuqian1212@163.com (S.L.);; 2 College of Petroleum Engineering, China University of Petroleum, Beijing 102249, China; 3 College of Energy Innovation, China University of Petroleum, Beijing 102249, China

**Keywords:** fracturing fluid leakoff, spurt loss, fluid-loss control, borate-crosslinked HPG, high-permeability reservoirs

## Abstract

Borate-crosslinked hydroxypropyl guar (HPG) gels are widely used as water-based fracturing fluids in oilfield stimulation. During hydraulic fracturing, their effectiveness depends on the rapid formation of a low-permeability filter cake on fracture walls, which helps reduce fluid invasion, maintain fracture pressure, and support fracture propagation. In high- and ultra-high-permeability reservoirs, however, rapid matrix invasion may occur faster than effective filter-cake formation, causing severe pre-cake spurt loss or even uncontrolled leakoff. Conventional filter-paper tests tend to emphasize stabilized wall-building behavior and may therefore fail to represent the early-time spurt loss in porous reservoir media. In this study, the leakoff behavior of borate-crosslinked HPG fracturing fluids was investigated using a modified static fluid-loss apparatus. Experiments were conducted at differential pressures of 0.5–6.0 MPa through filter paper and artificial sandstone disks with permeabilities from 0.120 to more than 4.0 μm^2^. The filter-paper tests showed typical wall-building behavior, with limited spurt loss and stable late-time leakoff. In contrast, the sandstone-disk tests revealed a transition from cake-controlled leakoff to early-time spurt-loss-dominated leakoff as permeability and differential pressure increased. When permeability exceeded approximately 1.55–2.42 μm^2^, spurt loss (*V*_sp_) became the main contributor to total leakoff, whereas the late-time wall-building coefficient (*C*_w_) was much less sensitive to permeability. This indicates that permeability mainly controls the pre-cake invasion stage rather than the stabilized leakoff stage. Based on these results, an empirical spurt-loss model considering permeability and pressure differential was developed, and spurt-loss zoning maps were constructed for engineering evaluation. Limited ultra-high-permeability tests further showed that quartz particles promoted early bridging and reduced leakoff under moderate pressure differentials, but the particle-assisted barrier lost effectiveness under higher pressure differentials. These findings demonstrate that filter-paper-based criteria are insufficient for evaluating HPG gel performance in extreme-permeability formations and that a spurt-loss-based framework is needed for fluid-loss-control design and fracturing-fluid selection in high-permeability reservoirs.

## 1. Introduction

**Figure 1:**
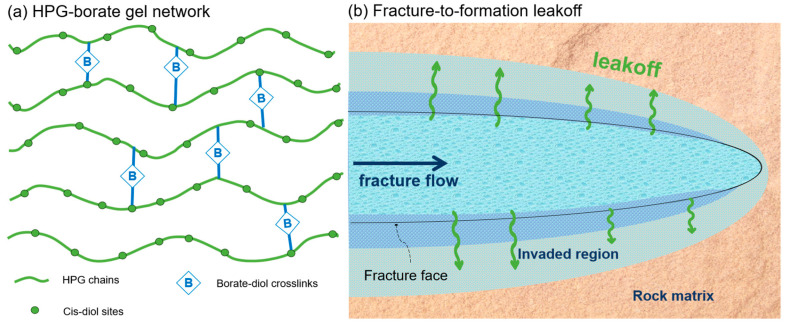
Conceptual framework of borate-crosslinked HPG gel leakoff in high-permeability sandstone. (**a**) Gel network crosslinking; (**b**) Fracturing-fluid leakoff into porous sandstone.

Fluid leakoff is a controlling variable in hydraulic fracturing because it governs pressure maintenance, fracture growth, fluid efficiency, and the amount of polymer available for proppant transport [1]. Guar gum and its derivatives, particularly hydroxypropyl guar (HPG), are widely used in water-based fracturing fluids because they hydrate efficiently and can be crosslinked into elastic hydrogels [2,3]. In fracturing operations, fluid intrusion into the formation was formally studied by Howard and Fast [4] and bounded as the leakoff of fracturing fluid. Crosslinked gels lose water (dehydrate) on the fracture surface under differential pressure [5]. The fluid entering a porous rock matrix is termed filtrate. The dehydrated gel builds up on the fracture wall to form a filter cake layer. For the borate-crosslinked HPG system studied here, its structure and leakoff process are shown in Figure 1. The leakoff cannot be treated only as a macroscopic flow problem. It should be considered as a coupled process involving gel-network structure, porous-media filtration, particle retention, and filter-cake formation.

Classical leakoff analysis separates early spurt loss from late-time wall-building leakoff. After an external filter cake is established, cumulative leakoff often follows a nearly linear relationship with the square root of time. The slope of this region is expressed as the wall-building leakoff coefficient, *C*_w_, and the intercept or equivalent early leakoff volume is expressed as spurt loss, *V*_sp_. This framework is useful when gel-cake formation is faster than fluid invasion into the matrix. However, this assumption becomes uncertain in high- and ultra-high-permeability sandstone [6]. Large pore throats and high pressure differentials can allow borate-crosslinked HPG fluids, filtrate, and fine particles to enter the near-surface pore network before a continuous cake develops [7]. In this case, the late-time *C*_w_ may appear acceptable after cake formation, but *V*_sp_ may already account for a large fraction of the total leakoff. Severe leakoff reduces the efficiency of fracturing treatment (leakoff volume of fracturing fluid reaches 90%) and poses potential challenges [8]. The pressure maintenance within the fracture becomes complex as a result. There have been some successful fracturing operations in unconsolidated sandstone reservoirs, but they do not always meet expectations [9,10,11]. The selection of fracturing fluid mainly relies on fluid familiarity [12]. Therefore, the timely formation of an effective filter cake layer during injection and the accurate quantification of gel leakoff are particularly crucial for unconsolidated sandstone reservoirs. Standard filter-paper tests are therefore insufficient by themselves for predicting core-scale leakoff in high-permeability sandstone, because they promote rapid and uniform cake formation under idealized filtration conditions.

Previous studies have improved the understanding of fracturing-fluid leakoff from several directions. Early work established the roles of filter-cake resistance, pressure differential, fluid rheology, and additives in modifying leakoff behavior [13,14,15]. Later studies extended the Carter-type description by considering non-Newtonian fluids, radial leakoff, pressure-dependent leakoff, and variable filter-cake properties [16,17]. Experimental studies also showed that polymer concentration, slurry viscosity, particle size, pore structure, and saturation conditions can strongly affect cake formation and leakoff-front development in porous media [18,19,20]. Recent work on HPG-based gels further linked crosslinking chemistry, reinforcement additives, and gel structure to rheological and filtration performance [3,21,22,23].

However, most early experimental frameworks were confined to rock cores with permeabilities below 1 μm^2^ [24,25]. In unconsolidated sandstone or extreme-permeability environments, the massive fluid loss presents a severe challenge for characterizing gel filtration [12]. Consequently, researchers such as Nguyen et al. [26] shifted away from static macroscopic coefficients, proposing pressure-dependent leakoff (PDL) models to better capture dynamic invasion, though these theoretical models often require stronger experimental validation.

Pressure-dependent leakoff (PDL) studies address a related but different problem. PDL is usually formulated at the fracture/reservoir scale to interpret pressure behavior, fracture geometry, fissure opening, pressure-sensitive permeability, or pressure-dependent fluid exchange between a hydraulic fracture and the surrounding formation. These models are useful for pressure-transient interpretation and hydraulic-fracture simulation, but they do not directly resolve the laboratory-scale competition between HPG-gel invasion into Darcy-level sandstone and external filter-cake formation. Therefore, the present study does not propose a complete PDL formulation. Instead, it develops a pressure- and permeability-dependent spurt-loss correction within the Carter-type wall-building framework.

Concurrently, innovative experimental setups have advanced the micro-mechanistic understanding of gel filtration. Reddy [18] utilized precision ceramic disks to conduct compositional analyses of retained cakes. Lü et al. [27], Liu et al. [28], and Sanchez Reyes et al. [29] explored the pressure-responsiveness of crosslinked gels, revealing that while elevated pressures enhance filtration driving forces, they simultaneously induce stress-compaction that improves the anti-filtration capacity of the gel layer. Direct observations of gel-cake morphology and thickness were achieved by Ma et al. [30] using customized micro-visual setups, while Brattekås et al. [13] tracked the dynamic penetration front of gels in multiphase porous media. More recently, the role of composite structures has gained attention: Wang et al. [19] demonstrated that introducing appropriately sized particulates significantly densifies the resulting filter cake, and Li et al. [20] elucidated the shear-thickening behavior and morphological evolution of viscoelastic polymers injected into sand-based porous media.

Overall, significant work remains in quantifying the leakoff behavior of fracturing fluid in reservoirs with different permeabilities. This study involves conducting leakoff experiments using artificial rock cores with a broad range of permeabilities, especially including ultra-high permeability conditions. The paper aims to elucidate the influence of leakoff and provide a foundation for selecting appropriate fracturing fluids for unconsolidated sandstone formations.

This study does not simply report additional static fluid-loss tests. The scientific novelty of this work is threefold. First, the experimental permeability range was extended to Darcy-level and ultra-high-permeability sandstone, including full-diameter sandstone disks of 2.42 μm^2^ and exploratory reduced-area disks exceeding 4.0 μm^2^. Second, the study separates early pre-cake spurt-loss-dominated invasion from late-time Carter-type wall-building leakoff for borate-crosslinked HPG gels in high-permeability sandstone. The results show that high permeability does not necessarily eliminate wall building: once an external filter cake is established, the late-time *V* − *t*^1/2^ relation remains approximately linear, and *C*_w_ is only weakly dependent on permeability. The main leakoff risk occurs before cake stabilization, where pressure-driven matrix invasion sharply increases *V*_sp_. Third, the proposed *V*_sp_ (*k*, Δ*p*) correlation provides a laboratory-calibrated screening model for early spurt-loss risk in high-permeability sandstone. This model should be regarded as a pressure- and permeability-dependent spurt-loss correction within the Carter-type framework.

## 2. Results and Discussion

### 2.1. Filter Paper Leakoff Experiments

**Figure 2:**
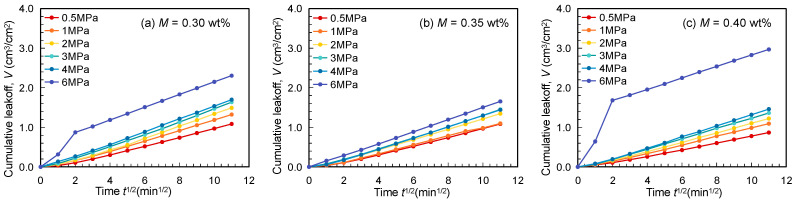
Representative filter-paper cumulative leakoff curves for crosslinked HPG fluids with different HPG concentrations, *M*: (**a**) 0.30 wt%, (**b**) 0.35 wt%, and (**c**) 0.40 wt%.

Filter-paper tests showed typical wall-building leakoff behavior across all three HPG concentrations. As shown in Figure 2, cumulative leakoff (*V*) scaled linearly with the square root of time (*t*^1/2^) over the measured period. This trend indicates rapid external cake formation and stabilized late-time leakoff controlled by the filter cake. These results validate the applicability of classical leakoff theory to the tested HPG systems under ideal conditions, establishing a baseline for subsequent core-scale comparisons.

**Figure 3:**
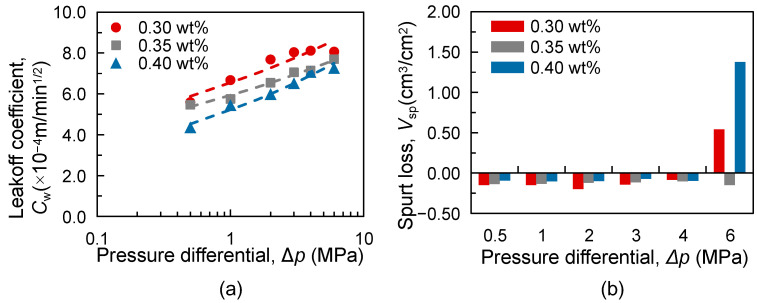
Filter-paper leakoff parameters at different HPG concentrations and pressure differentials. (**a**) Wall-building leakoff coefficient (*C*_w_). (**b**) Spurt loss (*V*_sp_).

Straight-line segments in these cumulative curves were fitted to obtain the slopes and intercepts. The corresponding wall-building leakoff coefficient *C*_w_ and apparent spurt loss *V_sp_* for 18 sets of filter paper leakoff tests are summarized in Figure 3. At a constant polymer concentration, *C*_w_ increased with pressure differential. This reflects the stronger filtration driving force at higher pressure. Conversely, under identical pressure differential, *C*_w_ decreased as HPG concentration increased. This directly reflects that more concentrated hydrogels enhanced network density and wall-building capacity, thereby reducing late-time leakoff. This pressure-dependent leakoff response aligns with the compaction mechanisms proposed by Mayerhofer et al. [31] under different test conditions.

Notably, *V*_sp_ remained close to zero at 0.5–4.0 MPa, becoming definitively positive only at the highest tested pressure (6.0 MPa). Slightly negative fitted values should be regarded as regression artifacts rather than physical negative spurt loss. This confirms that the uniform pore structure of filter paper facilitates nearly instantaneous gel-cake establishment, so the pre-cake invasion stage was very short under most tested conditions.

For each polymer concentration, the pressure dependence of *C*_w_ (m/min^1/2^) follows a power-law model.

(1) $$C_{w}=a\Delta p^{n}$$

where *a* is the fitted coefficient, Δ*p* is the pressure differential, MPa. And *n* is the pressure exponent. As detailed in Table 1, the fitted values of *n* range from approximately 0.14 to 0.20, with the coefficient a strictly on the order of 5–7 × 10^−4^.

Assuming complete polymer retention within the filter cake, the theoretical filtration coefficients satisfy the mass-balance boundary conditions.

(2) $$\frac{C_{w}}{C_{w}^{\prime }}=\sqrt{\frac{M^{\prime }}{M}}\times \frac{\Delta p^{n}}{\Delta p^{\prime n}}$$

where Superscript prime indicates experimental data.

Because the standard static fluid-loss test is commonly evaluated at a pressure differential of 3.5 MPa [32], the fitted relations were verified at this pressure. The calculated *C*_w_ values agreed with the measured values within approximately 5% (referring to $C_{w}^{0.3\%\text{-}3.0MPa}=7.69\times 10^{-4}m/\min^{1/2}$), as shown in Table 2. This agreement indicates that the power-law relation can reproduce the late-time filter-paper leakoff coefficient within the tested pressure range.

The filter-paper tests accurately captured typical late-time wall-building leakoff behavior for the borate-crosslinked hydrogels. Within 0.5–6.0 MPa, the late-time leakoff data showed an approximately linear relationship between cumulative leakoff and $t^{1/2}$. The apparent spurt loss remained small, with all values below 2 cm^3^/cm^2^. This behavior shows that filter paper promotes rapid filter-cake formation and is suitable for evaluating leakoff after cake stabilization. The pressure-dependent increase in *C*_w_ is consistent with the behavior expected from classical filtration theory. However, this idealized medium artificially restricts spurt loss, confirming its inability to represent pre-cake invasion dynamics. As demonstrated in our subsequent core tests, especially at higher permeability and pressure differential, early-time spurt loss can increase sharply and account for a large fraction of total leakoff. Relying solely on filter-paper criteria implicitly neglects this dominant pre-cake invasion, inevitably causing severe underestimations of total hydrogel leakoff in actual reservoirs.

### 2.2. Core Tests: Transition from Cake-Controlled Leakoff to Early-Time Spurt Loss Dominated by High Permeability

**Figure 4:**
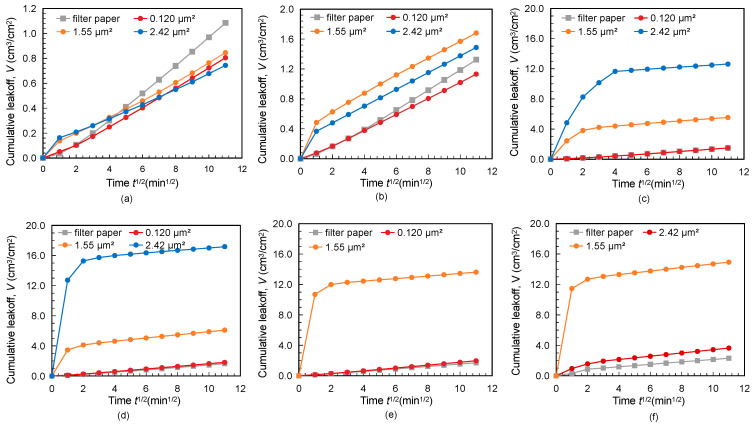
Cumulative leakoff curves of 0.30 wt% HPG gels for filter paper and sandstone disks (0.120–2.42 μm^2^) under different pressure differentials: (**a**) 0.5 MPa, (**b**) 1.0 MPa, (**c**) 2.0 MPa, (**d**) 3.0 MPa, (**e**) 4.0 MPa, and (**f**) 6.0 MPa.

Core leakoff tests were performed on sandstone disks with permeabilities of 0.120, 1.55, and 2.42 μm^2^. The results show that the dominant leakoff response changed as the pressure differential increased as shown in Figure 4. At low pressure differentials, leakoff was mainly controlled by rapid gel-cake formation. At higher pressure differentials, early-time fluid invasion became more important, and spurt loss increased with core permeability. This transition was reflected by a steeper initial leakoff segment, a measurable spurt-loss volume, and a delayed establishment of the late-time linear leakoff regime compared with the filter-paper tests.

At low pressure differentials, 0.5–1.0 MPa, all core curves were close to the filter-paper curves (Figure 4a,b). The late-time *V* − *t*^1/2^ relationship remained approximately linear, and *V*_sp_ was close to zero. Within this pressure range, increasing matrix permeability from 0.120 to 2.42 μm^2^ produced only a little measurable change in leakoff behavior. These results indicate that leakoff was governed mainly by rapid gel-cake growth and cake resistance, rather than by core permeability. Consequently, under mild pressure differentials, the filter-paper-derived wall-building coefficient can be used as an approximate late-time coefficient for these sandstone cores. This approximation is not valid for the early spurt-loss stage or for higher pressure differentials.

The response changed when the pressure differential reached 2 MPa or above. At 2–3 MPa (Figure 4c,d), the 0.120 μm^2^ core still showed an almost linear V − $t^{1/2}$ trend similar to that of the filter paper. In contrast, the 1.55 and 2.42 μm^2^ cores showed steeper early-time segments. This indicates greater initial leakoff before an effective gel cake was steady. The effect became stronger as both pressure differential and permeability increased. This behavior suggests that, at higher pressure differentials, fluid invasion into the connected pore network increasingly competes with surface cake formation, thereby promoting spurt loss.

At 4–6 MPa, differences among cores were more pronounced (Figure 4e,f). The 0.120 μm^2^ core deviated slightly from the filter-paper response even at 6 MPa, whereas the 1.55 μm^2^ core displayed substantial early-time divergence. For the 2.42 μm^2^ core at 6 MPa, most leakoff occurred within the first minute, preventing a reliable determination of the late-time wall-building coefficient. The progressive steepening of early-time curves indicates that fluid invasion outpaced cake building, and spurt loss became the dominant early-time behavior in higher-permeability cores.

Overall, for cores below 3 μm^2^, low-pressure leakoff can be reasonably represented by filter-paper-derived wall-building parameters. At higher pressures, especially in more permeable cores, early-time leakoff becomes highly sensitive to permeability and spurt loss. Direct application of filter-paper coefficients to core-scale analysis under these conditions is unreliable.

### 2.3. Wall-Building Leakoff Coefficient Across Core Permeabilities

Across all core experiments, cumulative leakoff curves stabilized within approximately 16 min, showing a linear correlation between leakoff volume *V* and the square root of time *t*^1/2^ (Figure 4). This indicates effective filter-cake formation in most samples. Exceptions occurred in high-permeability cores (*k* = 2.42 μm^2^) at Δ*p* > 4 MPa, where rapid early fluid loss prevented stable cake development. These observations confirm that the wall-building leakoff concept remains valid for interpreting late-time leakoff, provided the cake has stabilized.

**Figure 5:**
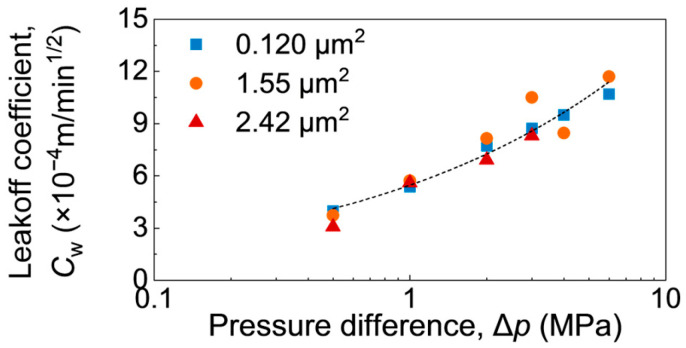
Pressure dependence of the late-time wall-building leakoff coefficient, *C*_w_. Comparison across core permeabilities.

Figure 5 summarizes the pressure dependence of the late-time wall-building leakoff coefficient *C*_w_ across cores of different permeabilities. Unlike early-time leakoff (spurt loss), *C*_w_ shows only weak sensitivity to permeability. Under identical pressure differentials, values of *C*_w_ were comparable across cores (e.g., 5.37–5.60 × 10^−4^ m·min^−0.5^ at Δ*p* = 2 MPa for *k* = 0.120–2.42 μm^2^). This confirms that once the filter cake is established, the late-time wall-building-controlled regime is largely independent of permeability.

The empirical relationship between *C*_w_ and Δ*p* can also be expressed as Equation (1). Where *a* = 4–6 × 10^−4^ m·min^−1/2^⋅MPa^-*n*^ and *n* = 0.3–0.5. Specific calculations are shown in Table 3.

### 2.4. Empirical Spurt-Loss Model and Engineering Zoning

**Figure 6:**
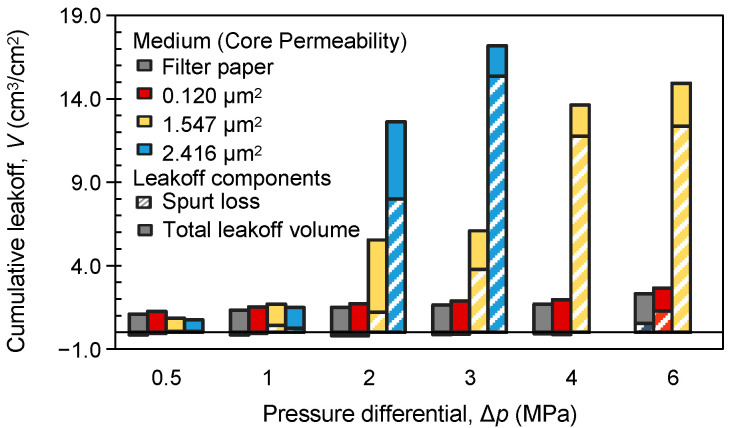
Pressure dependence of total leakoff and spurt loss for filter paper and sandstone disks with different permeabilities.

The spurt loss (*V*_sp_) and total leakoff volume (*V*_t_) exhibited similar trends across pressure differentials (Δ*p*), as shown in Figure 6. It reflected early-time fluid invasion before the filter cake reached dynamic equilibrium. At Δ*p* ≤ 1 MPa, *V*_sp_ remained near zero (<0.5 cm^3^/cm^2^) for all samples, indicating rapid cake stabilization. In contrast, at Δ*p* > 1 MPa, high-permeability rocks (*k* = 1.55–2.42 μm^2^) showed elevated *V*_t_ (1.5–17.2 cm^3^/cm^2^), which directly correlated with increased *V*_sp_. In permeable media, a large share of total fluid loss occurred before a stable external filter cake was established. Thus, spurt loss is the main source of total leakoff volume in highly permeable reservoirs. Some negative values were also observed under low-permeability and low-pressure conditions. These values should be interpreted as near-zero filtration caused by experimental uncertainty or data processing, rather than as physically meaningful negative spurt loss.

At a given permeability, a larger pressure differential accelerated both spurt loss and cake formation, leading the stable linear stage to appear earlier, as shown in Figure 4c,d. Higher permeability, corresponding to larger pore throats, further facilitates fluid entry. This behavior is reflected in lower effective entry pressures and higher spurt loss.

The empirical model developed in this section is intended to describe the pre-cake spurt-loss stage rather than the late-time wall-building leakoff stage. The response variable is the experimentally determined spurt loss, *V*_sp_, obtained from the late-time *V* − *t*^1/2^ extrapolation described in Section 4.5. The model was calibrated using the full-diameter sandstone-disk tests of 0.30 wt% HPG fluid within *k* = 0.120–2.42 μm^2^ and Δ*p* = 0.5–6.0 MPa. Filter-paper tests were not included in the parameter estimation because they do not contain a rock-permeability term and they strongly suppress early spurt loss. The reduced-area ultra-high-permeability tests and quartz-particle-additive tests were not used for fitting because their filtration area, leakoff state, and bridging mechanism differ from those of the calibrated full-diameter core tests.

**Figure 7:**
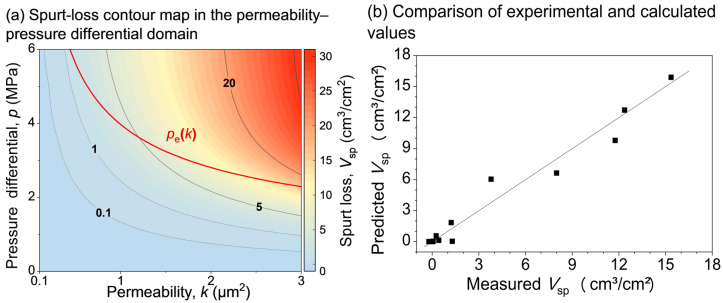
Internal calibration check and engineering application of the empirical spurt-loss model. (**a**) Spurt-loss contour map in the permeability–pressure differential domain calculated using Equation (5). (**b**) Comparison between experimentally determined and calculated *V*_sp_ values. The 1:1 line represents perfect agreement.

To quantitatively describe the initial leakoff behavior, it is necessary to adopt a model that satisfies two key conditions observed in Figure 6: (1) spurt loss increases nonlinearly with both pressure differential and core permeability, and (2) the initial spurt loss approaches a limiting value under high-pressure conditions. These features are naturally captured by a Hill-type empirical formulation, which has been widely used to describe saturating response phenomena in porous media. The proposed model is therefore expressed as:

(3) $$V_{\mathrm{sp},\mathrm{cal}}=Ak^{m}\frac{\Delta p^{n}}{\Delta p^{n}+{\left(Bk^{-1/2}\right)}^{n}}$$

where *V*_sp,cal_ denotes the calculated spurt loss, cm^3^/cm^2^; *k* is the core permeability, μm^2^; Δ*p* is the pressure differential, MPa; *A* is the limiting initial spurt-loss coefficient under high pressure; *m* is the permeability sensitivity exponent; *B* is the effective entry-pressure coefficient; and *n* controls the steepness of the pressure response. Model parameters, ***θ*** = (*A*, *m*, *B*, *n*)^T^, were obtained by minimizing the weighted sum of squared residuals, *S*(***θ***), using the Levenberg–Marquardt algorithm, as defined in Equation (4). The calculated *V*_sp_ values were compared with the experimentally determined *V*_sp_ values, and the final model achieved R^2^ = 0.969 and RMSE = 0.917 cm^3^/cm^2^, indicating that the model captures the nonlinear increase in spurt loss with permeability and pressure differential within the calibrated range. A predicted-versus-measured comparison was added in Figure 7b, where the 1:1 reference line indicates perfect agreement. These statistics indicate internal regression performance for the calibrated laboratory dataset; they are not independent field-scale validation and should not be interpreted as replicate-based uncertainty estimates.

(4) $$S(\theta )=\sum_{i=1}^{N}w_{i}{\left[V_{\mathrm{sp},\mathrm{obs},i}-V_{\mathrm{sp},\mathrm{cal},i}(\theta )\right]}^{2}$$

The final fitted equation for calculating the initial spurt loss is expressed as follows:

(5) $$V_{sp}=7.91k^{1.27}\frac{\Delta p^{4}}{\Delta p^{4}+{\left(3.97k^{-1/2}\right)}^{4}}$$

where *V*_sp_ is in cm^3^/cm^2^, *k* is the core permeability in μm^2^, and Δ*p* is the pressure differential in MPa. The model can be further interpreted using two characteristic quantities:

- *V*_max_(*k*), the limiting initial spurt loss under high-pressure conditions for a given permeability, calculated as *V*_max_(*k*) = 7.91 *k*^1.27^;
- Δ*p*_e_(*k*), the characteristic pressure differential, which also serves as the half-activation pressure differential at *V*_sp_ = 0.5 *V*_max_(*k*), given by Δ*p*_e_(*k*) = 3.97 *k*^−1/2^ MPa.

*V*_max_(*k*) and Δ*p*_e_(*k*) provide practical pressure scales for the onset and magnitude of early-time spurt loss. The experimental and modeling results indicate that core permeability primarily governs the magnitude and duration of early-time spurt loss, while pressure differential acts as the driving force amplifying this effect. In ultrahigh-permeability cores under high pressure differential, spurt loss can dominate the entire experimental window, and a stable, linear late-time leakoff regime may not be fully established within the 121 min test period. This observation motivates the detailed evaluation of ultra-high-permeability cores in the following section.

To extend the spurt-loss model to reservoir suitability evaluation for hydraulic fracturing, the established empirical equation was used to calculate the critical pressure differential under different allowable spurt-loss limits. For engineering evaluation, three spurt-loss thresholds were adopted: *V*_sp_ = 1.0 cm^3^/cm^2^ (early-warning), 5.0 cm^3^/cm^2^ (high spurt-loss risk, aligned with industry standard [33]), and 20.0 cm^3^/cm^2^ (severe-loss threshold). The corresponding critical pressure differentials, Δ*p*_c_, were calculated.

Table 4 summarizes the calculated characteristic Δ*p*_e_ and critical pressure differentials Δ*p*_c_. As permeability increases, both Δ*p*_e_ and Δ*p*_c_ decrease, narrowing the safe pressure differential window. For low-permeability cores, spurt loss remains limited even under elevated pressure differential. In contrast, for cores with *k* > 2.42 μm^2^, high-risk and severe-loss thresholds can be reached at a relatively low pressure differential. This result does not imply that high-permeability reservoirs cannot be fractured. Rather, it shows that their allowable pressure differential window is much narrower. Stronger fluid-loss control is therefore required in ultra-high-permeability reservoirs.

According to the industry standard [33], the spurt loss of wall-building water-based fracturing fluids should not exceed 5.0 cm^3^/cm^2^. Accordingly, *V*_sp,std_ = 5.0 cm^3^/cm^2^ was adopted as the standard control limit in this study. A heat map of the empirical spurt-loss function was plotted in Figure 7. Within the calibrated range, the characteristic pressure-differential curve Δ*p*_e_(*k*) passes through the transition interval bounded by *V*_sp_ = 1.0 and 20.0 cm^3^/cm^2^ and lies close to the standard threshold. This indicates that Δ*p*_e_(*k*) serves not only as a fitted pressure-scale parameter, but also as a practical engineering indicator for the onset of significant spurt loss.

**Figure 8:**
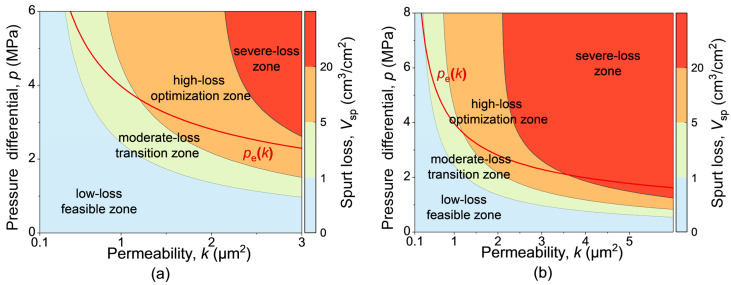
Engineering spurt-loss zoning maps for 0.3 wt% borate-crosslinked HPG fluid. (**a**) Zoning map within the calibrated permeability range. (**b**) Extrapolated zoning map for higher-permeability conditions.

Following these criteria, a spurt-loss engineering zoning map was constructed for a 0.3 wt% borate-crosslinked HPG fluid in Figure 8. The zoning consists of four regions:

- Zone 1 (*V*_sp_ = 1.0 cm^3^/cm^2^). Low-loss feasible zone; spurt loss is limited and conventional fracturing is applicable. Early-warning line at *V*_sp_ = 1.0 cm^3^/cm^2^, indicating spurt loss begins to increase noticeably.
- Zone 2 (1.0 < *V*_sp_ ≤ 5.0 cm^3^/cm^2^). Moderate-loss transition zone; early-time spurt loss begins to increase; Δ*p*e(*k*) indicates onset of significant spurt-loss growth.
- Zone 3 (5.0 < *V*_sp_ ≤ 20.0 cm^3^/cm^2^). High-loss optimization zone; strong spurt loss may reduce fluid efficiency; fluid-system and treatment design optimization required. The line at *V*_sp_ = 20.0 cm^3^/cm^2^ marks the severe-loss boundary.
- Zone 4 (*V*_sp_ ≥ 20.0 cm^3^/cm^2^). Severe-loss zone; stronger fluid-loss-control measures or alternative fracturing-fluid systems are required.

This zoning framework allows for straightforward evaluation of reservoir suitability. *V*_sp_ = 1.0 cm^3^/cm^2^ defines the early-warning threshold, indicating the Δ*p* at which spurt loss begins to increase. *V*_sp_ = 5.0 cm^3^/cm^2^ marks high spurt-loss risk, corresponding to the industry control limit for conventional water-based fluids. *V*_sp_ = 20.0 cm^3^/cm^2^ represents severe spurt loss, identifying high-risk reservoirs unsuitable for direct treatment with the base fluid. Using the empirical model, the spurt-loss risk map was extended to higher-permeability domains. Although the predicted spurt-loss magnitude may deviate from the 4.0 μm^2^ core experimental data, the predicted failure regimes align well with observed trends.

### 2.5. Exploratory Reduced-Area Disk Tests and Effect of Particle Additives

#### 2.5.1. Experimental Optimization and Feasibility

Fluid leakoff in ultrahigh-permeability rock occurs rapidly. Because each test was limited to a total fluid volume of 400 g, conventional rock disks were prone to complete fluid depletion during the experiment. To avoid this problem, small rock slices with reduced leakoff area were used in this study.

**Figure 9:**
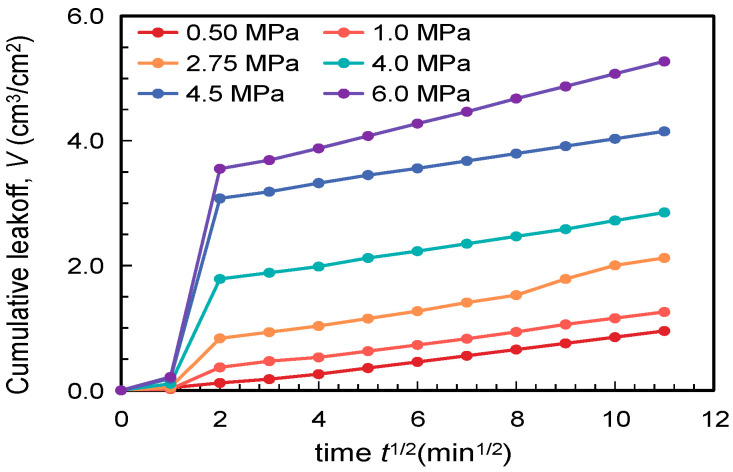
Cumulative leakoff curves for 1 μm^2^-permeability samples under different Δ*p*. Validates reduced-area design feasibility.

Feasibility tests were conducted on approximately 1 μm^2^-permeability samples under different pressure differentials. The cumulative leakoff curves entered a stable late-time regime within 16 min as shown in Figure 9. This result shows that the reduced-area design captured the early rapid leakoff stage and the subsequent late-time response within the available fluid capacity. The reduced-area disk tests were used as exploratory experiments to keep the leakoff volume within the measurable range and to evaluate whether a late-time filtration response could still be observed under ultra-high-permeability conditions. Because the perimeter-to-area ratio is larger for 25 mm disks, possible edge or wall-channeling effects cannot be excluded by area normalization alone. Therefore, these tests are used mainly to support qualitative interpretation of ultra-high-permeability leakoff behavior.

**Figure 10:**
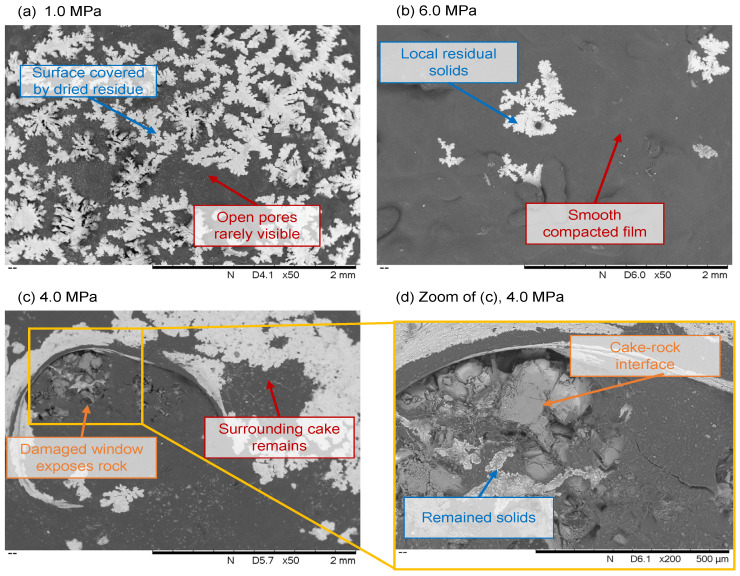
Post-test SEM images of the dried inlet surface of approximately 1 μm^2^-permeability rock disks tested under different pressure differentials. (**a**–**c**) At 1.0, 6.0, and 4.0 MPa, respectively, a dense filter-cake layer completely covered he core surface. (**d**) Zoom of (**c**), showing the cake–rock interface and remaining solids.

Post-test SEM observations support this interpretation, as shown in Figure 10. After drying, the inlet surfaces of the 1 μm^2^-permeability samples were largely covered by continuous solid residues. Open pores and original grain boundaries were rarely visible. Local damaged areas revealed that retained solids were mainly deposited at the inlet face, forming a relatively continuous external layer (Figure 10c). The enlarged view of the cake–rock interface shows compacted solids filling the near-surface pores (Figure 10d). These images, although representing dried post-test morphology rather than in situ leakoff, provide strong evidence that filter cakes formed effectively on the rock surface under the tested pressure differential conditions.

#### 2.5.2. Leakoff Characteristics in Ultra-High-Permeability Samples (4.0 μm^2^ Case)

Severe leakoff occurred under these ultra-high-permeability conditions. For the 0.3 wt% HPG fluid, cumulative leakoff reached nearly 87% of the initial fluid charge even at a pressure differential of 0.5 MPa. Increasing polymer concentration or adding fluid-loss-control particles reduced leakoff in some tests. This effect weakened at higher pressure differentials. These results indicate that conventional empirical leakoff descriptions based on stable surface-cake control are not suitable for this permeability range.

**Figure 11:**
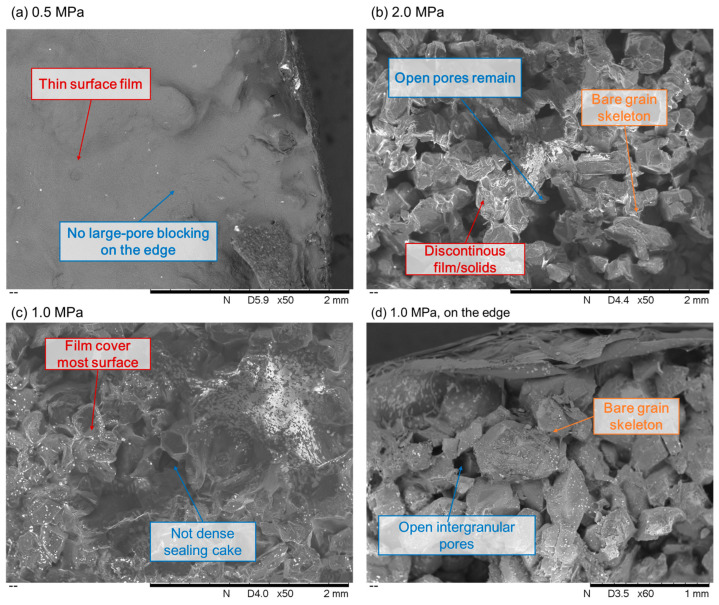
Post-test SEM images of the dried inlet surface of 4 μm^2^-permeability samples. (**a**) At 0.5 MPa, the inlet face was covered by a filter cake, and open pores were not exposed. (**b**–**d**) At 1.0 and 2.0 MPa, no continuous surface film was formed.

For the same 0.3 wt% HPG fluid, the dried inlet surface after testing is shown in Figure 11. Unlike the approximately 1 μm^2^-permeability samples, the 4.0 μm^2^ samples did not form a dense and continuous external filter cake at higher pressure differentials. The original pore structure remained partly visible. Retained solids appeared mainly as local patches along pore walls, pits, and pore throats (Figure 11).

**Figure 12:**
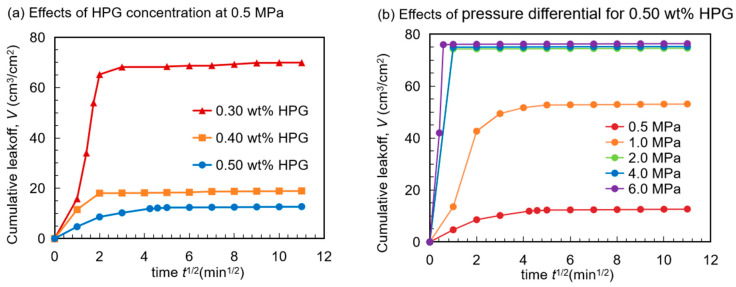
Effects of (**a**) HPG concentration and (**b**) pressure differential on cumulative leakoff in approximately 4.0 μm^2^-permeability samples.

This morphology indicates local bridging and shallow internal deposition, rather than complete surface sealing. The large pore-throat system allowed filtrate and fine particles to invade the near-surface pores before a stable cake could form. This interpretation is consistent with the leakoff curves, where most fluid was lost before the late-time response stabilized in several high-pressure tests (Figure 12). Leakoff failure in these samples was therefore controlled by early invasion and delayed cake formation, not by the final resistance of a mature filter cake.

(1) Mechanism of Polymer Concentration and Pressure Differentials

Increasing the HPG concentration from 0.30 wt% to 0.50 wt% reduced the total leakoff volume, but it did not ensure rapid filter-cake stabilization (Figure 12). The higher polymer concentration increased fluid viscosity and promoted the retention of polymer-rich material near the inlet face. However, the deposited layer still needed time to densify into a stable, low-permeability barrier. A higher polymer concentration therefore improved leakoff resistance, but it did not fully eliminate the early spurt-loss stage.

The pressure differential controlled whether this barrier could form in time. At moderate pressure differentials, retained material accumulated at the inlet face and gradually formed an effective filter cake. At higher pressure differentials, the early flow surge invaded the pore network before a stable cake was established. Under this condition, increasing HPG concentration mainly improved the late-time resistance of the deposited layer. It could not fully suppress early spurt loss. Therefore, the benefit of polymer concentration was limited by the competition between pressure-driven invasion and filter-cake formation.

(2) Role and Pressure Limit of Quartz Particles

**Figure 13:**
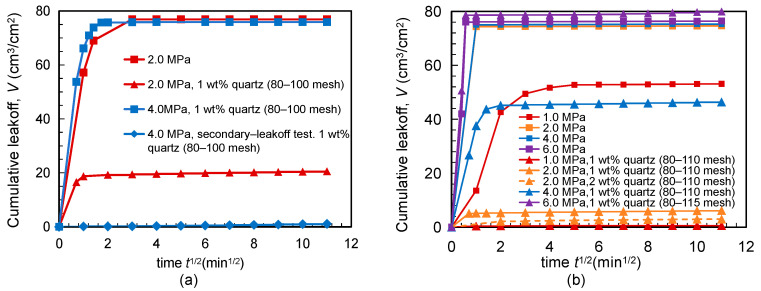
Effect of quartz-particle addition on cumulative leakoff under different pressure differentials for (**a**) 0.30 wt% HPG concentration and (**b**) 0.50 wt% HPG.

Quartz particles improved leakoff control by promoting early bridging. In the 0.30 wt% HPG system, adding 1 wt% quartz reduced cumulative leakoff from 87.5% to 25.75% at 2.0 MPa (Figure 13a). This large reduction indicates that quartz particles were retained near large pore entrances and provided a framework for rapid filter-cake development. The effect was strongly pressure-sensitive. At 4.0 MPa, cumulative leakoff again exceeded 90%, even with quartz addition. The particle bridge was therefore unstable under stronger pressure-driven invasion. It was likely penetrated, displaced, or bypassed before a continuous external cake could form. The reinjection test supports this interpretation. After a near-complete breakthrough during the initial 4.0 MPa test, reinjection through the same sample showed a stable low-leakoff trend. This result indicates that retained material could control later filtration once a cake had formed. The main failure occurred during the initial spurt-loss stage, before the sealing structure was established.

For the 0.50 wt% HPG system, quartz particles expanded the controllable filtration window (Figure 13b). At pressure differentials up to 2.0 MPa, the particle-assisted system suppressed early invasion and maintained low cumulative leakoff. At 4.0 MPa, quartz addition still reduced total leakoff compared with the particle-free fluid. At approximately 6.0 MPa, however, the particulate structure lost stability and severe leakoff occurred again.

These results show that quartz particles reduce spurt loss but cannot eliminate it in ultra-high-permeability rocks. Their main function is to establish an early bridging framework before pressure-driven invasion penetrates the near-surface pore network. When the invasion rate exceeds the rates of particle retention and cake formation, the additive loses effectiveness. Particle addition should therefore be designed for rapid early-stage bridging. In rocks with very large pore throats, some initial leakoff may be unavoidable. The key is to convert this early leakoff into stable wall building before excessive fluid loss occurs.

(3) Summary of Leakoff Mechanisms in Ultra-High-Permeability Cores

**Figure 14:**
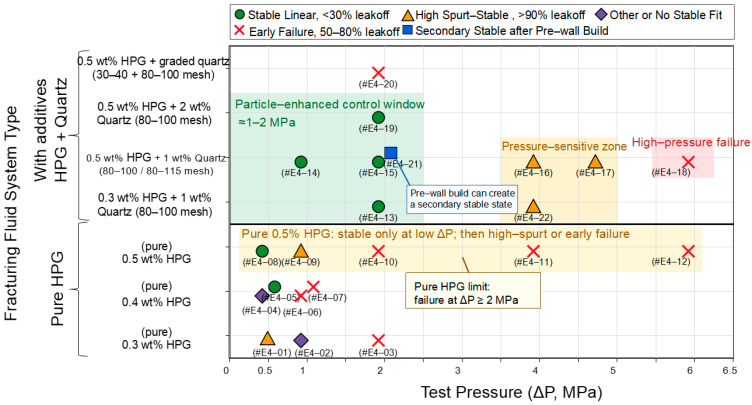
State classification of leakoff behavior in ultra-high-permeability cores: stable linear filtration, high-spurt followed by stable filtration, and premature breakthrough. The labels in parentheses, such as #E4-01, denote the corresponding experimental run IDs.

Based on the series of tests on approximately 4.0 μm^2^ samples, leakoff behavior can be classified into three states: stable square-root-time filtration, high-spurt followed by late-time stabilization, and premature breakthrough (Figure 14). These states reflect different balances between pressure-driven invasion and filter-cake formation.

Leakoff control in ultra-high-permeability rocks is governed by a kinetic competition between the invasion rate and the wall-building rate. Permeability controls the pore-throat scale and the depth of early invasion. Pressure differential controls the driving force for that invasion. Under high permeability and high pressure differential, early-time invasion can dominate the whole test. In such cases, a stable late-time leakoff stage may not appear within the 121 min experimental window.

Increasing HPG concentration improves fluid viscosity, particle suspension, and final cake resistance. However, it cannot suppress severe spurt loss when pressure-driven invasion is faster than cake formation. Quartz particles promote early bridging and expand the stable filtration window at moderate pressure differentials, especially at 1.0–2.0 MPa. Their effect is limited at higher pressure. At 4.0 MPa, the particle bridge becomes pressure-sensitive and may be penetrated or bypassed. Near 6.0 MPa, the bridge can fail, and severe leakoff occurs again.

Therefore, fluid-loss-control design for ultra-high-permeability formations should not rely only on increasing polymer concentration or solid loading. The key is to build a stable barrier before excessive fluid is lost. Future optimization should focus on rapid early-stage bridging, particle retention near large pore throats, and pressure-resistant filter-cake structures under high differential pressure.

## 3. Conclusions

This study examined the leakoff behavior of borate-crosslinked HPG fracturing fluids in high- and ultra-high-permeability sandstone media. The main conclusions are as follows.

1. The standard filter-paper test reproduced typical wall-building leakoff behavior, but it underestimated early-time leakoff in high-permeability sandstone. It is suitable for comparing stabilized filter-cake performance. It should not be used alone to evaluate fracturing fluids for high-permeability reservoirs. Within the tested range, the wall-building leakoff coefficient increased with pressure differential and decreased with increasing polymer concentration.
2. Core experiments showed that the dominant leakoff mechanism changed with permeability and pressure differential. In cores with permeability below approximately 2.5 μm^2^ and at low pressure differential, leakoff was mainly controlled by the stabilized filter cake. The response was close to the filter-paper result. In higher-permeability cores, substantial fluid invasion occurred before an effective cake was established. Early-time spurt loss and late-time stabilized leakoff should therefore be evaluated separately.
3. The empirical pressure-permeability analysis translates the experimentally observed separation between early spurt loss and late wall-building leakoff into a *V*_sp_(*k*,Δ*p*)-based screening framework. The proposed zones identify the pressure–permeability conditions under which early spurt loss, rather than stabilized wall-building leakoff, becomes the controlling leakoff risk. The proposed spurt-loss-based zones provide a practical screening method for identifying low-leakoff, transition, high-risk, and severe-leakoff conditions within the tested range. They should be treated as engineering correlations for the tested borate-crosslinked HPG system, not as universal criteria.
4. Ultra-high-permeability tests confirmed the limited pressure tolerance of particle-free HPG systems. Increasing polymer concentration improved leakoff control, but it did not eliminate early invasion or breakthrough. Quartz particles reduced leakoff only when they were retained near pore entrances and formed an early bridging structure. At high pressure differential, this structure was penetrated, bypassed, or destabilized, and severe leakoff occurred again. Leakoff control in ultra-high-permeability sandstone is therefore governed by the combined effects of viscosity, pressure differential, permeability, particle size, and particle retention.

The correlations obtained in this study are specific to the tested borate-crosslinked HPG system, artificial sandstone media, and laboratory geometry. Further work should examine natural unconsolidated sandstone, elevated temperature, dynamic filtration, and broader particle-size distributions, and repeated tests using permeability-matched cores to quantify experimental uncertainty.

## 4. Materials and Experimental Methods

### 4.1. Materials

The fracturing fluids used in this study were based on hydroxypropyl guar (HPG). The HPG powder and the borate-based crosslinker consistent with those used in Bohai Oilfield field operations were employed. All chemical reagents were used as received without further purification.

**Figure 15:**
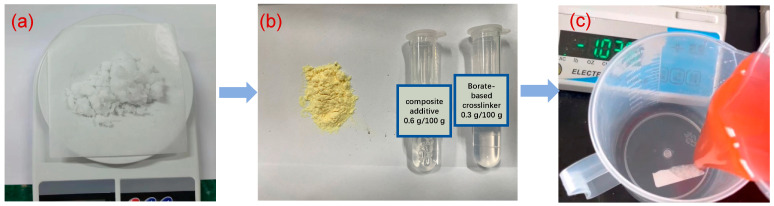
Schematic of fracturing-fluid preparation: (**a**) weighing sea salt for artificial seawater preparation; (**b**) preparing the fracturing fluid; (**c**) crosslinked fracturing fluid.

Artificial seawater was prepared by dissolving pre-weighed sea salt crystals (supplied by the Blue Coral Sea Crystal Processing Factory, Hangu, Binhai New Area, Tianjin, China) in water, as shown in Figure 15a. Under continuous stirring, the HPG powder (Figure 15b) was gradually added to the artificial seawater to ensure uniform dispersion. After the polymer was fully hydrated at constant temperature, a neutral color indicator was introduced, followed by dropwise addition of the borate-based crosslinker to initiate gel crosslinking. The resulting crosslinked fracturing fluid exhibited noticeable wall-hanging behavior during pouring, indicating successful gel formation, as illustrated in Figure 15c.

In selected experiments, quartz sand with a particle size of 30–120 mesh was added to the fracturing fluid as a fluid-loss control additive. The sand was uniformly dispersed in the fluid prior to filtration experiments to promote filter-cake development on the filtration surface. The quartz sand was not intended to simulate proppant transport, but to evaluate its influence on early-time leakoff behavior and wall-building performance.

The overall fluid preparation procedure followed standard practices recommended by the International Organization for Standardization [34], ensuring that the obtained fluid properties are representative of commonly used field formulations. Multiple fracturing fluid formulations with different polymer concentrations were prepared to investigate the influence of fluid properties on leakoff behavior. The viscosity characteristics of the prepared fracturing fluids are summarized in Table 5. All fluids were prepared at ambient temperature and allowed sufficient time for hydration and crosslinking prior to filtration experiments.

### 4.2. Filtration Media: Filter Paper and Artificial Sandstone Disks

**Figure 16:**
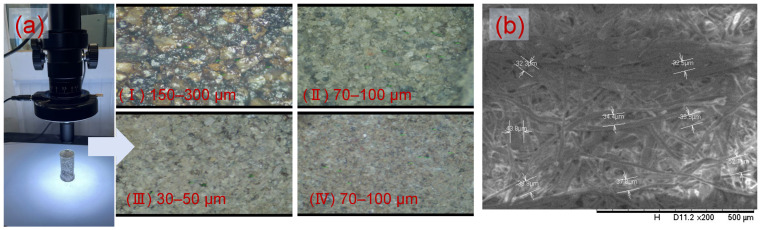
Pore-scale characteristics of filtration media: (**a**) pore sizes of natural cores observed by optical microscopy; (**b**) SEM image of filter paper.

The filtration media consisted of specialized filter paper and artificial core materials. Natural sandstone cores display pore sizes of 30–300 μm, whereas the experimental filter paper exhibits a narrower pore size range of 30–50 μm, as shown in Figure 16. Artificial sandstone disks based on a quartz sand–epoxy resin system were used as filtration media.

Artificial sandstone disks were prepared to cover a permeability range from approximately 0.120 to 4.0 μm^2^, representing moderate-, high-, and ultra-high-permeability conditions. To maintain consistency with standard filter paper tests, disks with a diameter of 50 mm were used for samples with permeability below approximately 3 μm^2^, providing an effective filtration area comparable to that of the filter paper. For samples with higher permeability, additional experiments were conducted using disks with a reduced diameter of 25 mm to obtain measurable leakoff data under constant differential pressure conditions. Area normalization was used to express leakoff per unit exposed area, but it should not be interpreted as proof that the two disk sizes are fully equivalent. The reduced-diameter disks were used only for exploratory ultra-high-permeability tests because full-diameter disks could cause near-complete depletion of the available fluid charge before the late-time leakoff response could be evaluated.

The measured permeabilities of the artificial sandstone disks are summarized in Table 6 for 50 mm-diameter disks. Within each permeability group, the filtration area was kept constant to ensure consistency when comparing leakoff behavior under different experimental conditions.

### 4.3. Static Fluid-Loss Experimental Apparatus

**Figure 17:**
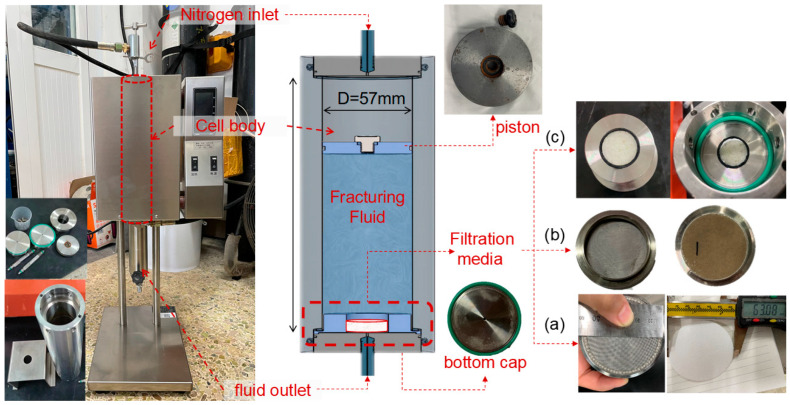
Static leakoff apparatus and sample configurations: (**a**) filter paper; (**b**) full-diameter rock disk with the same diameter as the filter paper; (**c**) reduced-diameter rock disk for ultra-high-permeability tests.

An improved static fluid-loss apparatus, modified from a commercial static fluid-loss apparatus supplied by Kence Instrument Co., Ltd. (Shanghai, China), was developed to assess leakoff behavior in high-permeability samples. The system configuration is illustrated in Figure 17, which presents both the complete apparatus and different filter media assemblies.

The filtration section was configured to accommodate (a) filter paper, (b) artificial sandstone disks with a diameter comparable to that of the filter paper, and (c) reduced-diameter artificial sandstone disks for ultra-high-permeability samples. This modular configuration ensured that the same apparatus could be used consistently across all experiments while accommodating differences in permeability and sample geometry. In the sandstone-disk tests, the disk circumference was sealed with a sealing ring and sealing tape to prevent side-wall bypass flow. The fluid was therefore designed to pass through the exposed disk surface in the axial direction.

A constant differential pressure was applied across the filtration medium using compressed gas. Pressure was monitored throughout each experiment to ensure stable operating conditions. Filtrate passing through the filtration medium was collected continuously, and cumulative filtrate volume was recorded as a function of time.

### 4.4. Experimental Procedure

Prior to filtration experiments, artificial sandstone disks were fully saturated to eliminate the influence of trapped gas on leakoff measurements. Saturation was achieved using a negative-pressure saturation method. Each disk was placed under vacuum conditions and subsequently submerged in the saturating fluid until complete saturation was ensured.

For each experiment, the selected filtration medium (filter paper or artificial sandstone disk) was installed in the filtration section of the apparatus. The prepared fracturing fluid was then introduced into the fluid chamber, and a predetermined differential pressure was applied across the filtration medium. Each test was conducted for a total duration of 121 min, with filtrate volume recorded at 11 time points corresponding to *t* = n^2^ min (n = 1–11).

The documented experimental program was larger than the subset used for quantitative regression. In total, the program included 18 filter-paper tests and more than 57 numbered rock-sample runs. Runs showing pressure-gauge instability, early blowout, near-complete leakoff before a stable late-time regime, or no interpretable linear segment were not used to build the principal quantitative correlations. They were retained as exploratory observations because they define the operating-risk boundary and the feasibility of leakoff-control measures. Therefore, the equations reported here should be interpreted as laboratory-scale engineering correlations within explicit validity ranges, not as universal constitutive laws.

### 4.5. Leakoff Parameter Determination

The amount of leakoff volume is generally of the form *V_t_/A = V*_sp_ *+ Ct*^1/2^ + γ*t*. Usually, because the filter cake builds up rapidly, the cake-accumulation stage in the actual cross-flow is often neglected in detail. The equation is simplified to *V_t_/A = V*_sp_ *+ Ct*^1/2^, *C = 2C*_w_. Since the static leakoff coefficient is not governed by dynamic shear rate [34], it is obtained by conventional static experimental tests.

**Figure 18:**
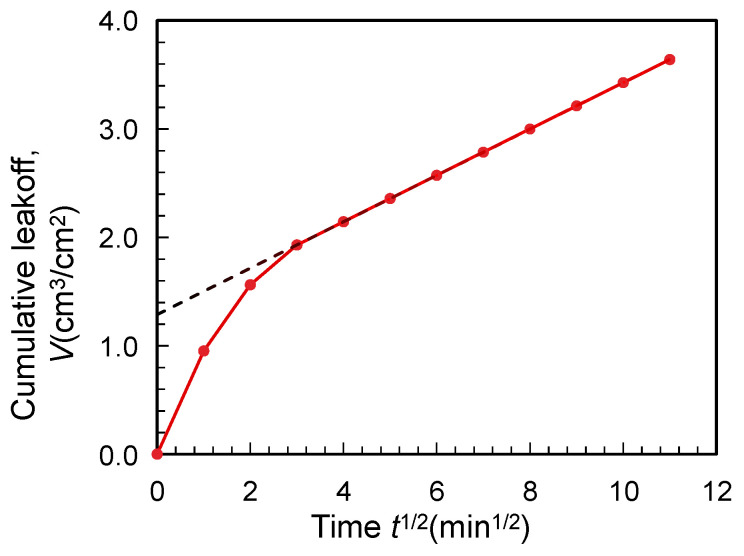
Leakoff parameter definition.

Leakoff behavior was quantified using conventional parameters commonly employed in static fluid-loss analysis. Because the effective filtration area differed between some of the media, the measured filtrate volume was normalized by area and expressed as leakoff per unit area, *V* (cm^3^/cm^2^). The total leakoff volume *V*_t_ was therefore converted according to *V* = *V_t_*/*A*, where *A* is the exposed filtration area. Leakoff behavior was interpreted by plotting *V* against the square root of time. When a stable linear segment was observed, the data were represented in the conventional form *V* = *Ct*^1/2^ + *b*. The graphical definition used for *C*_w_ (m/min^1/2^) and spurt loss *V*_sp_ (cm^3^/cm^2^) is illustrated in Figure 18. The leakoff curves shown in the figures represent individual valid runs or regression-derived values, not averages of duplicate or triplicate measurements. Because permeability-matched sandstone disks were limited and each test altered the inlet surface by cake deposition and polymer/filtrate invasion, replicate-based standard deviations and error bars were not calculated.

The wall-building leakoff coefficient, *C*_w_, was obtained from the slope of the late-time linear fit, whereas the intercept b was taken as the equivalent spurt-loss volume, *V*_sp_. In this paper, *V*_sp_ is used as an engineering descriptor of the early-time fluid loss prior to an effective external wall-building resistance being established. It does not imply a separate mechanistic stage. For experiments in which a clear linear segment could not be identified within the test duration, spurt loss was estimated based on the deviation of the early-time filtrate volume from the extrapolated late-time trend.

## Figures and Tables

**Table 1:** Fitted leakoff coefficients for filter-paper experiments.

Mass Concentration, *M* (wt%)	Parameter, *a* (m·min^−1/2^⋅MPa^−n^)	Pressure Difference Index, *n*	R-Square, R^2^
0.30	6.54 × 10^−4^	0.154	0.877
0.35	5.93 × 10^−4^	0.143	0.983
0.40	5.21 × 10^−4^	0.204	0.972
Average	5.89 × 10^−4^	0.165	0.976

**Table 2:** Verification of *C*_w_ calculation results at 3.5 MPa.

Mass Concentration, *M* (wt%)	Predicted Value (m/min^1/2^)	Measured Values (m/min^1/2^)	Error
0.30	8.25 × 10^−4^	8.25 × 10^−4^	−0.03%
0.35	7.14 × 10^−4^	7.30 × 10^−4^	−2.16%
0.40	6.39 × 10^−4^	6.13 × 10^−4^	4.21%

**Table 3:** Calculation results of leakoff coefficients for core tests below 3 μm^2^.

Permeability, *k* (μm^2^)	Parameter, *a* (m·min^−1/2^·MPa^−*n*^)	Pressure Difference Index, *n*	R-Square, R^2^
0.120	5.58 × 10^−4^	0.380	0.984
1.55	5.79 × 10^−4^	0.392	0.871
2.42	4.98 × 10^−4^	0.479	0.956
All	5.47 × 10^−4^	0.410	0.919
All (No cake compressibility)	4.94 × 10^−4^	0.500	0.885

**Table 4:** Characteristic and critical pressure differential for selected spurt-loss thresholds.

*k* (μm^2^)	*V*_max_ (cm^3^/cm^2^)	Δ*p*_e_ (MPa)	Δ*p*_c_ (*V*_sp_ = 1 cm^3^/cm^2^) (MPa)	Δ*p*_c_ (*V*_sp_ = 5 cm^3^/cm^2^) (MPa)	Δ*p*_c_ (*V*_sp_ = 20 cm^3^/cm^2^) (MPa)	Engineering Interpretation
0.120	0.54	11.46	N.R.	N.R.	N.R.	Negligible spurt loss
0.50	3.28	5.61	4.57	N.R.	N.R.	Low spurt loss risk
1.00	7.91	3.97	2.45	4.55	N.R.	*V*_sp_ begins to be significant
1.55	13.8	3.19	1.69	2.77	N.R.	Rapid rise in *V*_sp_ after Δ*p*_e_; consistent with experiments.
2.42	24.3	2.55	1.16	1.82	3.76	Severe loss at high Δ*p*; 4 MPa above Δ*p*_c_ (*V*_sp_ = 20 cm^3^/cm^2^), fluid nearly completely filtered; consistent with experiments.
3.000	31.9	2.29	0.97	1.50	2.61	High-risk extrapolated case
4.000	46.0	1.99	0.76	1.17	1.86	Severe-loss risk at low pressure differential
6.000	77.0	1.62	0.55	0.83	1.25	Poor fluid-loss control expected

Note: N.R. means that the threshold is not reached within the model prediction range because *V*_max_ < *V*_sp_.

**Table 5:** The viscosity characteristics of fracturing fluids with different guar gum concentrations. (Base solution viscosity before crosslinking: 27 mPa·s at 25 °C).

	HPG Powder, wt%	Viscosity at 25 °C, mPa·s	Viscosity at 60 °C, mPa·s
#1	0.30	666	30.4
#2	0.40	925	255
#3	0.50	963	372

**Table 6:** Permeability and porosity of artificial sandstone disks (50 mm diameter).

Sample ID	Porosity (%)	Permeability (μm^2^)
#P1	15.0	0.120
#P2	18.1	1.55
#P3	17.3	2.42

## Data Availability

The original contributions presented in this study are included in the article. Further inquiries can be directed to the authors.

## Abbreviations/Nomenclature

Nomenclature	
Symbol	Description
*A*	Exposed filtration area (cm^2^)
*a*	Model parameters
*B*	Model fit coefficient
*b*	Intercept of the *V*–*t*^1/2^ plot (spurt loss)
*C*	the slope of the linear *V*–*t*^1/2^ region (m/min^1/2^)
*C*_w_	Wall-building leakoff coefficient (m/min^1/2^)
*k*	Formation permeability (μm^2^)
*M*	Polymer (gel) concentration of fracturing fluid
m	Model exponent controlling permeability
*n*	Model exponent controlling pressure dependence
*p*	Pressure difference (MPa)
Δ*p*_c_	Critical pressure corresponding to a specified leakoff *V*_c_ (MPa)
Δ*p*_e_(*k*)	Characteristic pressure (half-leakoff pressure) (MPa)
*S(**θ**)*	Weighted least-squares objective function
*t*	Time (min)
*V*	Leakoff per unit area (cm^3^/cm^2^)
*V*_t_	Total leakoff volume (cm^3^)
*V*_sp_	Spurt loss (cm^3^/cm^2^)
*V*_sp__,__cal_	Calculated value of model spurt loss (cm^3^/cm^2^)
*V*_sp__,__std_	Critical spurt loss in industry standards (cm^3^/cm^2^)
*V*_max_	Maximum spurt loss predicted by the empirical model (cm^3^/cm^2^)
*w_i_*	Weighting factor for the *i* data point
***θ***	Parameter vector, (A,m,B,n)^T^
γ	Dynamic filtration correlation coefficient
Δ*p*	Experimental leakoff pressure difference (MPa)
Abbreviations	
HPG	hydroxypropyl guar
PDL	pressure-dependent leakoff
R^2^	Coefficient of determination
RMSE	Root mean square error (cm^3^/cm^2^)
